# Innate and Adaptive Immune Systems in Physiological and Pathological Pregnancy

**DOI:** 10.3390/biology12030402

**Published:** 2023-03-03

**Authors:** Jessica Weng, Camille Couture, Sylvie Girard

**Affiliations:** 1 Mayo Clinic Medical Scientist Training Program, Mayo Clinic Graduate School of Biomedical Sciences, Mayo Clinic Alix School of Medicine, Rochester, MN 55905, USA; 2 Department of Microbiology, Infectiology and Immunology, Universite de Montreal, Ste-Justine Hospital Research Center, Montreal, QC H3T 1C5, Canada; 3 Department of Obstetrics & Gynecology, Department of Immunology, Mayo Clinic, Rochester, MN 55905, USA

**Keywords:** pregnancy, innate immune system, adaptive immune system, immunological tolerance, maternal–fetal interaction, immune cells, physiological pregnancy, pathological pregnancy

## Abstract

**Simple Summary:**

Pregnancy is a continually evolving process with many moving parts that must be tightly regulated on both the maternal and fetal sides to ensure its success. A significant component that must be appropriately regulated is the immune system, which is both an innate and adaptive key player at every stage of pregnancy. Pregnancy plays a unique role in the health outcomes of mothers and neonates later in life. Thus, there is a significant clinical and scientific need to understand the changes in innate and adaptive immune systems in physiological and pathological pregnancy to understand how these systems might be dysregulated in complications such as preterm birth, preeclampsia, and recurrent spontaneous abortions. This review will provide a comprehensive understanding of implicated immune cells and their intricate network of interactions in both normal and complicated pregnancies and highlight some challenges and outstanding questions that need further investigation.

**Abstract:**

The dynamic immunological changes occurring throughout pregnancy are well-orchestrated and important for the success of the pregnancy. One of the key immune adaptations is the maternal immune tolerance towards the semi-allogeneic fetus. In this review, we provide a comprehensive overview of what is known about the innate and adaptive immunological changes in pregnancy and the role(s) of specific immune cells during physiological and pathological pregnancy. Alongside this, we provided details of remaining questions and challenges, as well as future perspectives for this growing field of research. Understanding the immunological changes that occur can inform potential strategies on treatments for the optimal health of the neonate and pregnant individual both during and after pregnancy.

## 1. Introduction

Dr. Peter Medawar, in the 1950s, asked the fundamental question of how pregnancy is possible [1]. The fetus possesses paternal antigens, is semi-allogeneic, and has a gestational length of 9 months, during which both the maternal and fetal innate and adaptive immune systems are active. Thus, Medawar later hypothesized three mechanisms that allow for a successful pregnancy. Anatomic separation of mother and fetus is no longer a leading theory since the placenta is involved in the controlled bi-directional exchange of maternal and fetal material. Antigenic immaturity of the fetus as the second mechanism is no longer a leading theory since the exchange of material across the placenta has shown to activate immune cell populations in both the mother and fetus [2]. The third proposed mechanism, which has garnered the most evidence, is the immunologic tolerance of the mother toward the fetus.

Throughout gestation, events such as implantation and placentation, fetal growth, and parturition are distinct processes requiring a unique immune environment. Different immunological profiles are needed at specific times during pregnancy, and the success of pregnancy depends on the ability of the maternal immune system to adapt successfully [3]. Early in pregnancy, the local profile at the maternal–fetal interface is predominantly proinflammatory, which then shifts to an anti-inflammatory state to promote development and tolerance, and returns to a proinflammatory phenotype at the initiation of labor [3]. For example, implantation is initiated when the trophoblast cells make their way through the surface epithelial lining of the decidua to attach and invade, leading to the formation of the placenta. Pregnancy success depends on the coordinated balance between the invading trophoblast and a receptive maternal decidua, where immune cells represent a major cellular component. The post-implantation decidua is rich in immune infiltrates comprising innate and adaptive immune cells [4,5]. Nearly 70% of decidual leukocytes are natural killer (NK) cells, 20–25% are macrophages (M Φ), and approximately 1.7% are dendritic cells (DC); approximately 3–10% are T cells, and B cells are present but in low numbers [4,5,6,7,8,9]. Additionally, less than 5% of CD45^+^ immune cells in the decidua are neutrophils [10]. In a physiological setting, the immune responses at the maternal–fetal interface support the blastocyst’s reception, establishment, and growth. Immune cells are crucial for pregnancy success, and depleting them from the implantation site, can lead to pregnancy loss [11,12]. Additionally, when these immune cells are dysregulated later in pregnancy, it can lead to pregnancy complications such as preterm birth (PTB) and preeclampsia (PE) and contribute to uncontrolled inflammation, negatively affecting placental function and fetal development [13,14,15,16,17,18,19,20]. Besides depletion, the imbalance of immune cells, their polarizations, and functional changes may also lead to pathological pregnancies.

Though tolerant of fetal antigens, pregnant individuals can still mount robust immune responses [21]. For this, the maternal and fetal immune cells have established a cooperative status, allowing the innate and adaptive immune systems to protect the mother and fetus from external insults such as pathogens. In response to infectious diseases, the innate immune system relies on pattern recognition receptors to detect specific bacterial/viral structures. Innate immunity is the first line of defense but cannot provide specific immunity that prevents reinfection. Instead, the innate immune system utilizes a limited number of innate proteins and receptors; these are inherited from an individual’s parents and do not need to be generated de novo, allowing it to detect and destroy invaders within minutes to hours [22]. Furthermore, members of the innate immune system will present antigens to the adaptive immune system to initiate the second wave of protection.

The adaptive immune system relies on a repertoire of antigen receptors to recognize structures specific to individual pathogens, providing it with greater sensitivity and specificity. Adaptive immune responses, however, tend to be slower and predominantly involved in more chronic complications of pregnancy, such as PE, recurrent spontaneous abortion (RSA), chronic villitis of unknown etiology (VUE), and more. These chronic inflammatory conditions mostly rely on adaptive immune cells: subsets of B and T cells are of particular interest in framing the adaptive immune system response in physiological and pathological pregnancy.

Much remains to be learned about how the innate and adaptive immune systems work together throughout gestation in both physiological and pathological pregnancy. Furthermore, levels of hormones are known to change tremendously during pregnancy and their impact on the immune system have recently been reviewed extensively [23,24,25]. Therefore, this will not be a focus of the current review. With the breakdown of cell populations within and across the two immune systems in physiological and pathological pregnancies, this review paper hopes to start bridging that gap. For this review, we will focus on the most prominent cells of the innate (i.e., DC, monocytes/macrophages, neutrophils, and NK cells) and adaptive (i.e., B and T cells and their subtypes) with specific focus on maternal immune cells- and what is known about their roles in physiological and pathological pregnancies. This review will present our current understanding of how innate and adaptive immune responses contribute to pregnancy, generalize the key roles of each system, and address the immune switch from physiological to pathological in pregnancy.

## 2. Innate Immune System

Pregnancy is considered a unique immunological paradigm that requires the maternal immune system to protect the mother and fetus from infection throughout pregnancy while also orchestrating a successful pregnancy. The key innate immune cells found in the decidua and placenta include neutrophils, monocytes/MΦ, DC, and NK cells (the latter part of the innate lymphoid cells (ILCs)) [26]. These cells can directly and rapidly respond to host defense and play a critical role in further educating the adaptive response to induce tolerance. Due to their essential roles, dysfunction in these cell types can have significant repercussions; this section will focus on the role of the innate immune system in physiological and pathological pregnancies.

### 2.1. The Innate Immune System in Physiological Pregnancy

#### 2.1.1. Dendritic Cells

DC are essential antigen-presenting cells whose roles have been mainly debated due to subsets that are more fluid than once thought. As we learn more about DCs, their categorization has become increasingly complex and, at times, nebulous; therefore, we will discuss them as a single-cell type for the purpose of this review.

DCs interact closely with other immune components such as T cells (see Section 4), NK cells, and macrophages to keep a pregnancy-friendly environment [27,28,29]. Studies show that there is an intimate connection between immature DCs and NK cells in the first-trimester decidua, suggesting a pregnancy-specific interaction of these cells and their role in the local immune response [30,31]. The decidua, as compared to the endometrium of non-pregnant individuals, house the majority of DCs clustered in proximity with NK cells [30,31]. This proximity allows interactions between these cells that regulate their expansion and apoptosis to maintain an optimal balance for physiological pregnancy outcomes [31,32]. During pregnancy, a crucial role of DCs located at the maternal–fetal interface is avoiding the activation of T cells specific to inoffensive antigens or risk-inducing autoimmune disease. One way to prevent T cell activation and thus promote fetal immune tolerance is through DC entrapment within the decidua [33]. The regulatory functions of DCs during pregnancy go beyond simply promoting maternal tolerance to the fetus, impacting other processes such as decidualization and placentation through cell differentiation and the associated vascular adaptations mainly via their production of vasoactive mediators [12,33,34,35,36,37,38,39,40].

#### 2.1.2. Monocytes/Macrophages

Monocytes are bone-marrow-derived leukocytes that circulate in the peripheral blood and are characterized by their ability to recognize “danger signals” via pattern recognition receptors [41]. At the onset of physiological pregnancy, circulating monocytes will infiltrate the decidua, where they can differentiate into either MΦ or DCs [42]. As pregnancy progresses, there is a matched progressive increase in monocyte activation in the maternal circulation [43]. These monocytes can be classified into classical, intermediate, or non-classical subsets based on the expression of key surface markers, CD14 and CD16 [44]. MΦ also have polarizations status, namely pro-inflammatory (M1) and homeostatic (M2), with the latter including several subtypes (M2a, b, c) [45]. Decidual MΦ play key immune roles at the maternal–fetal interface due to their interactions with other immune cells and their secretion of cytokines, all of which are dependent on their polarization status. In physiological pregnancy, MΦ in the decidua adopt a predominantly anti-inflammatory and tolerant, known as M2, polarization status and decreased pro-inflammatory M1 [46]. This decreased M1/M2 ratio modifies the immune microenvironment to support and maintain the pregnancy and has been reviewed previously in-depth [47,48,49]. More work is needed to therapeutically target MΦ polarization clinically, but recent work has shown promising advances [50,51].

Early after conception, decidual MΦs are activated by the introduction of seminal fluid antigens, eliciting an inflammatory response that plays a crucial role in implantation [52,53]. As gestation progresses, these cells will change their overall polarization and are characterized by an immunosuppressive and tolerant phenotype, supporting the significant role of these cells in the maternal immune tolerance towards the fetus [42,54]. Throughout pregnancy, MΦs at the maternal–fetal interface display self-renewal capacities, play a critical role in the maintenance of tolerance to fetal antigens, and are key in the initiation of parturition and postpartum tissue repair [55]. In terms of labor initiation, monocytes/MΦs can be activated by soluble factors such as damage-associated molecular patterns (DAMPs), micro-particles, and cytokines released by the placenta [13,56,57,58]. Following their activation, monocytes/MΦs secrete proinflammatory cytokines (TNF-α, IL-12, IL-6, and IL-8), prostaglandins, enhance phagocytic activity, and produce reactive oxygen species, altogether contributing to successful parturition [55,59].

#### 2.1.3. Neutrophils

Neutrophils are the most abundant type of granulocyte and are first responders to sites of inflammation, making them a hallmark of acute inflammation [60,61]. During pregnancy, studies in humans and mice have found that neutrophils are recruited to the implantation site, where they can infiltrate the myometrium, fetal membranes, and cervix during labor, especially in cases with ascending infections [62,63]. Pregnant people have increased neutrophils in the circulation, but these neutrophils have decreased phagocytic activity and oxidative burst [64,65,66,67]. Their presence leads to increased levels of parturition-signaling proinflammatory cytokines in the fetal membranes, including IL-1β, IL-6, IL-8, and TNF-α [62,68]. Neutrophils also promote angiogenesis by upregulating their expression of VEGF-A and arginase-1 in the decidua early in pregnancy and play a role in tissue remodeling of the uterus and cervix in the pre- and post- partum period [62,69].

#### 2.1.4. Natural Killer Cells

NK cells play an essential role in detecting self versus non-self. They can detect and attack non-self through cytolytic activity and secrete cytokines and chemokines to propagate an inflammatory cascade that will lead to the recruitment of nearby immune cells [70]. NK cells are divided into subpopulations, namely uterine NK cells (uNK), peripheral blood NK cells (pNK), and decidual NK cells (dNK) based on their location. Until recently, uNK cells were thought to be equivalent to cytotoxic pNK cells, which play a role in the immune response against viruses, tumors, and infections. However, uNK cells are not implicated in a cytotoxic response against the embryo but rather can respond to hormones and have regulatory functions to ensure proper embryo implantation, trophoblast invasion, and placentation. This is carried out primarily through the facilitation of vascular remodeling and angiogenesis by trophoblasts through the release of IL-8 and IFN-inducible protein-10 (IP-10) [11,71,72,73,74,75,76,77,78]. uNK cells accumulate large numbers at the implantation site, have proximity to the extravillous trophoblasts, and have receptors that recognize trophoblast HLA, suggesting that they could regulate their invasion, which is essential for establishing the maternal blood supply to the fetus [79,80]. Additionally, the relationship between uNK cells and angiogenesis indicates that increased production of angiogenic factors translates into greater peri-implantation blood flow and, thus, an increase in the oxidative process [74,81]. Overall, the precise role of uNK cells in physiological pregnancy is still debated. dNK are the primary leukocytes in first-trimester decidua and have roles in trophoblast invasion and protection against infection. The proportion of these cells decreases throughout pregnancy but are still present in the term decidua [82,83]. dNK cells during pregnancy can express a senescent phenotype as they highly express inhibitor receptors KIR which interact with specific HLA ligands expressed on extravillous trophoblasts to dampen the cytotoxic capability of dNK cells [76,84,85]. This is one of the mechanisms through which HLA are promoting maternal immune tolerance of the fetus. In particular, this is carried out through HLA-G expression on extravillous trophoblasts which is a key modulator of dNK cells actions and involved in tolerance whilst preserving dNK cells activation [85]. The role of HLAs in maternal–fetal immune tolerance has been reviewed extensively [86,87].

### 2.2. The Innate Immune System in Pathological Pregnancy

#### 2.2.1. Dendritic Cells

In addition to the essential roles of DCs in physiological pregnancy, defined above, DCs have also been implicated in pathological pregnancies. The aberrant differentiation/maturation and functions of DCs may interrupt maternal–fetal tolerance and have been associated with several pregnancy complications, such as RSA, PTB, or PE [88,89,90,91]. For example, the activation of DC in a PTB model, i.e., lipopolysaccharide (LPS) injection in the mouse, suggests they play a role in the premature induction of labor [92]. Additionally, in patients with PE, we can see enhanced recruitment of DCs via chemokines such as granulocyte-macrophage colony-stimulating factor to the decidua and their increased maturation [93,94]. Besides their role in the maternal–fetal tolerance, disrupted uterine DC function has been associated with up- or down-regulation of the immune response following infection-related pregnancy complications in mice [95]. In many cases, the involvement of DCs in pathological pregnancies is related to their ability to communicate with and regulate the adaptive immune response, which will be discussed in Section 3 [96].

#### 2.2.2. Monocytes/Macrophages

Currently, the literature points to location-specific changes, namely maternal circulation vs. maternal–fetal interface, in the numbers of monocytes/MΦ in patients with pregnancy complications. When looking at maternal circulation, monocytes were elevated in PTB [48]. Regarding fetal monocytes isolated from cord blood, these were shown to be linked to the inflammatory profile of the placenta following PTB, whereby placental lesions were shown to be associated with gene expression changes in fetal monocyte subsets [97]. These placental lesions showed correlations to biological processes in monocytes, where acute placental inflammation correlated with enhanced monocyte activation, chemotaxis, and platelet function, but maternal vascular malperfusion lesions had negative correlations with these processes in monocytes [97]. There is more M1 polarization due to pro-inflammatory response at activation of the Notch signaling pathway [50,98].

In another complication of pregnancy, it has been reported that monocyte numbers were elevated in maternal circulation but that MΦ numbers were reduced in patients with PE compared to normal controls [99] However, Lockwood et al. reported a significant increase in the number of MΦ within the decidua of patients with PE [100]. In support, a study showed that in addition to having more decidual MΦ in patients with PE, there was a subsequent reduction in trophoblast invasion of the uteroplacental arteries and accumulations of apoptotic trophoblasts around arterial walls [101]. It has been postulated that this increase in MΦ is associated with a proinflammatory M1 polarization [102]. The increase in apoptotic trophoblasts may prevent the needed trophoblast invasion seen in physiological settings, which may lead to PE [103].

Similar findings have also been demonstrated in early pregnancy in patients with RSA. Decidual MΦs in these patients had decreased cathepsin E production, which plays essential roles in apoptosis, inhibition of angiogenesis, and increasing immune responses, which has been proposed to be associated with RSA [104]. Within the decidua, there is a skewed M1/M2 macrophage population [46]. Supporting the role of MΦs in this apoptosis cascade are studies showing that the proinflammatory cytokines secreted by MΦs increase Fas expression and enhance trophoblast sensitivity to Fas-mediated apoptosis [105,106]. When looking at changes in circulating levels of monocytes, it was discovered that a relative value of IL-10^+^ monocytes below 27.0% could predict pregnancy termination in patients with threatened early miscarriage and RSA [107]. Interestingly, elevation in both M1 and M2 subtypes of MΦ were observed within the placenta in cases of villitis of unknow etiology (VUE), with a predominance of M1 [108].

#### 2.2.3. Neutrophils

During pregnancies complicated with intraamniotic infection, neutrophils can be found in the amniotic fluid [109]. These neutrophils can be either of fetal or maternal origin or a mix of both, providing evidence that both the fetus and the mother participate in the host defense mechanisms against intraamniotic infection [109]. In addition, neutrophil invasion into the amniotic cavity indicates preterm labor onset [62,68,110,111]. However, work in rodent models has shown that systemic depletion of neutrophils before intraamniotic infection did not delay the onset of PTB, suggesting that their role in labor remains to be elucidated [112,113,114].

Locally, in the decidua, and systemically in the maternal circulation, neutrophil numbers increase throughout physiological pregnancy; however, in both locations, this increase is prominent in PE pregnancies, particularly those with early-onset PE [66,115,116,117]. Interestingly, even though neutrophil numbers have been shown to increase in PE, their phagocytic function decreased further compared to normal pregnancies [66]. In the maternal circulation, activated neutrophils can release inflammatory mediators, such as reactive oxygen species, TNF-α, or extracellular traps (NETs), which are associated with endothelial dysfunction, the latter of which is a hallmark of PE [61,118,119]. The activation of neutrophils is thought to occur partly from the micro-debris released from the placental syncytiotrophoblast, suggesting a possible role of neutrophils in PE [120].

#### 2.2.4. Natural Killer Cells

Work on the role of NK cells in pregnancy complications has been most evident in PE. The primary function of uNK cells, which is to coordinate with extravillous trophoblasts to remodel the spiral arteries, is limited in PE and key for the initiation of the pathology [121]. There is, however, a lack of consistent evidence regarding the number of NK cells in PE compared to normotensive pregnancies. Some studies report lower numbers of NK cells within the decidua in PE [122,123], while others report opposite findings [124,125]. Trophoblasts altered vascular conversion and insufficient invasion of the uterine lining are the primary defects in PE and disorders such as fetal growth restriction and RSA [126,127,128,129]. There is evidence of an NK shift in PE, as well as differences in the cytotoxicity of NK cells between patients with physiological pregnancies and patients with RSA, which could be exerted through their natural cytotoxicity receptors [130,131,132,133]. Differences have been found in the cytotoxicity of NK cells between patients with physiological pregnancies and patients with RSA [130,131]. Overall, the regulatory mechanisms of NK cells remain to be fully defined, and more work is needed, particularly related to NK cell function in association with their numbers.

## 3. The Adaptive Immune System in Pregnancy

In pregnancy, an individual can carry the fetus from conception to term without immune rejection, even though the fetus expresses paternal antigens creating a semi-allogeneic graft. The nine-month gestational period from implantation to birth allows for underlying maternal conditions and conditions that develop mid-gestation to intimately involve the adaptive immune system. As stated previously, the innate immune system must be tightly regulated to promote appropriate placental and fetal development. Immune dysregulations are strongly associated with pregnancy complications, and the same is valid for the adaptive immune system. Within the latter, the primary players are the B and T lymphocytes. When there is dysfunction or imbalance of T and B lymphocyte subtypes, there are important consequences resulting in RSA and PE, among other complications. Here, we will explore the functions of T and B lymphocytes and their subtypes in physiological and pathological pregnancy.

### 3.1. The Adaptive Immune System in Physiological Pregnancy

#### 3.1.1. T Cells

T cells are lymphocytes that play essential roles in the adaptive immune system; they can directly kill infected host cells, activate other immune cells, and produce cytokines to regulate the immune response. Since the fetus carries paternal antigens, the first question to answer is whether these lymphocytes are aware of these foreign antigens. Mouse studies have shown that maternal T cells are aware of paternal alloantigens and subsequently initiate a reduction in reactive T cells during pregnancy [134].

Maternal awareness of the foreign antigens in the fetus protects itself from attack in several ways. The fetus actively defends itself from attack by maternal T cells by catabolizing tryptophan via trophoblasts and macrophages, required for T cell proliferation [135] and reducing T-cell-dependent complement activation [136]. There has also been evidence that T cells specific to fetal antigens decrease in number and remain low postpartum [137]. Trophoblasts, the main parenchymal cell of the maternal–fetal interface, can also induce the Fas-mediated death of T cells as a support of peripheral clonal deletion tolerance [138]. The remaining clonotypic T cells also become unresponsive to antigenic stimulation. Furthermore, while studies have found no significant alteration of total T cells or absolute lymphocyte count during pregnancy or after birth [139,140], there are conflicting data on T cell subsets throughout gestation [141].

The Th1/Th2/Th17/Treg balance in the pregnant individual’s circulation is vital, and helper T cells play an essential role in the tolerance of the fetus through different strategies [142]. Successful pregnancies are generally correlated with a Th2-biased environment [143]. These Th2 cells secrete cytokines such as IL-4, IL-5, IL-6, IL-9, IL-10, and IL-13, which minimize inflammation and promote a tolerant environment promoting survival of the fetus. Th17 cells, which secrete IL-17 (a potent proinflammatory cytokine), are significantly lower in the third trimester of pregnancy than in non-pregnant individuals [144].

The maternal CD4^+^CD25^+^ regulatory T cell pool is also expanded in pregnancy; it is not driven by alloantigens and is essential for suppressing maternal immune attack against the fetus [145,146,147]. Several studies have found that exposure to the seminal fluid itself is the initial underlying mechanism of the expansion of tolerant T cells [148]. The adoptive transfer of Treg cells from a non-pregnant mouse has been shown to prevent fetal rejection [149]. Fetal rejection functions as graft-versus-host disease of the fetus [150,151,152]. The proportion of effector and naïve Treg cells increases in the peripheral blood from the first through to the second trimester of pregnancy and subsequently decreases postpartum [147,153]. These changes in proportions reflect the dynamics of feto-maternal tolerance that is still unclear to this day.

Besides the CD4^+^ helper T cells, it has been revealed that highly differentiated CD8^+^ effector memory cell sub-types are present at the human decidua [154]. Specifically, these decidual CD8^+^ cells may suggest some sort of recognition pathway. However, much is to be elucidated about this cell population and its purpose, especially in physiological pregnancy.

#### 3.1.2. B Cells

B cells are lymphocytes that play an essential role in humoral immunity and regulating immune homeostasis. In pregnancy, the role of B cells has been approached by examining the alterations in B cell population numbers and antibody production. B cells (CD19^+^CD20^+^) primarily decrease in normal pregnancy [141,155,156]. Although at least two B cell subsets have been reported, B1 and B2, there is not much known about their roles in physiological pregnancy. The CD5^+^ B1 cell subset decreases, suggesting a potential decrease in destructive autoantibodies [157].

Maternal antibodies serve a tremendous purpose in neonate immune protection immediately after birth and up to six months postpartum, but also play a role in regulating humoral immunity perinatally [158]. For example, lymphocytotoxic anti-paternal antibodies have been detected in normal pregnant individuals [159]. Further work found that the cytotoxic effects of lymphocytes against trophoblasts were decreased in the presence of maternal serum and recapitulated after removing IgG antibodies from maternal serum [160]. These anti-paternal IgG antibodies played a protective role in maternal tolerance of the fetus. Specific cytokines, such as IL-6, can signal placental B-cells to secrete more asymmetric IgG antibodies [161]. At this time, little work has been carried out on B cells and their functions within the placenta.

Furthermore, Kelemen et al. described a progesterone-induced blocking factor produced by the placenta, which also induces B cells to produce pregnancy-protective antibodies, the latter of which cannot activate immune effector responses [162]. Valeff et al. showed that B cell activation through the B cell receptor is lowered during pregnancy and, even with stimulation, produces lower levels of inflammatory cytokines at the transcriptomic level, suggesting that B cells are hypo-responsive during pregnancy [163]. This is, in part, another strategy for maternal tolerance of the fetus.

### 3.2. The Adaptive Immune System in Pathological Pregnancy

#### 3.2.1. T Cells

While the maternal and fetal immune systems work together to create a tolerant environment for the fetus, pathological pregnancy involves tissue rejection-like responses of the fetus. VUE is an inflammatory condition with infectious or non-infectious infiltration of maternal CD8^+^ T cells into the placenta. Several groups describe VUE as an allograft response against the fetus, such as graft-versus-host disease in transplant rejection [164,165,166]. Infiltration of maternal CD8^+^ T cells has also been identified in pathologies such as chronic chorioamnionitis, infiltration of maternal CD8^+^ T cells into the chorion, which ultimately can cause pregnancy complications such as PTB, preterm premature rupture of membranes (PPROM), and IUGR, among others [150,167]. Both chronic chorioamnionitis and VUE have similar chemokine and proinflammatory profiles [164,168].

As the tolerance of the semi-allograft from a T cell purview is multifactorial, an imbalance of T cell subtypes can manifest in RSA. A significant elevation of CD8^+^ cells in patients with RSA was found in the decidua [169]. A Th1-biased environment is also associated with RSAs, whereas Tim-3, which regulates Th1-cell-mediated immune response, expression is elevated in patients with RSA [170]. A reduction in Tregs also increases RSAs with Foxp3 as the transcription factor promoting the differentiation of naïve T cells to Tregs [171,172]. Lower expression of Foxp3, also regulated by STAT proteins, is involved in RSAs. Unfortunately, the dysregulation of various cell types and subtypes and a pro-inflammatory bias at the maternal–fetal interface leads to adverse pregnancy outcomes [173,174]. γ/δ T cells have been shown to have intimate involvement in RSA as well, with much lower progesterone-activated γ/δ T cells compared to normal pregnancies [175,176]. The Vδ2^+^ subtype was found to have significantly increased expression of CD107a with a higher proportion of IL-17A-secretion in the periphery (maternal circulation) compared to that of normal pregnancies [177]

As stated above, due to the dysregulation of DCs, Treg cells are decreased in PE with mechanisms involving Foxp3, similar to patients with RSAs [178]. Specifically, IL-17-producing T cells were significantly increased in patients with PE compared to healthy controls with additional higher levels of soluble endoglin, an inhibitor of TGF-β [144,179,180]. Other studies have added that the upregulation of Th17 cells contributed to PE along with the decreased Treg, which both modulate the Th1/Th2 immune balance, biasing a Th1 response [179,181].

#### 3.2.2. B Cells

Adverse pregnancy outcomes studied in antibody-mediated autoimmune diseases and pregnancy complications such as anti-phospholipid syndrome, RSA, PE, PTB, IUGR, and pregnancy-induced hypertension, are associated with a general increase in both the absolute and relative number of B cells [182,183,184,185,186,187,188]. Following these studies, the next question is the role of these increased B cells.

Since the fetus is semi-allogeneic, several groups have investigated the detrimental effects of autoantibodies in pathological pregnancy. For example, high levels of antiphospholipid antibodies, including lupus anticoagulant, anticardiolipin antibodies, and anti-beta2-glycoprotein-I have been associated with pregnancy complications such as fetal demise, spontaneous abortions, and PE [189]. Besides antiphospholipid antibodies, other autoantibodies, such as antibodies against the angiotensin II type I receptor, are strongly involved in the development of PE [190]. B1 cells that produced angiotensin II type I receptor when stimulated in vitro with serum from patients with PE [191]. Beard et al. showed that a lack of maternal cytotoxic antibodies led to RSA [192]. Asymmetric antibodies, protective IgG antibodies that do not make insoluble antigen-antibody complexes, are high in maternal serum titers. In contrast, they are significantly lower in pregnant individuals with RSA [193,194]. Thus, these asymmetric antibodies with relatively high affinity to the paternal antigen could play a protective role against RSA.

Jensen et al. found that patients with PE have sustained high CD19^+^CD5^+^ B cells, whereas patients with normal pregnancies have declining levels in the third trimester [191]. Besides autoantibodies, antibodies, in general, play a prominent role in pathological pregnancies. Patients with more than two abortions have higher positive sera with cytotoxic antibodies [195]. Much remains to be studied about the role of B cells, and more work is needed.

## 4. Innate Immune Interaction with Adaptive Immune Responses

Since innate immune cells play a critical role in educating and activating the adaptive response, we will explore how that control plays out during pregnancy.

DCs are a great example of this due to their active role in pregnancy to maintain a favorable environment and accomplish this through intimate interactions with T cells. Specifically, DCs can migrate to draining lymph nodes and present processed antigen peptides to T cells in an immune-stimulatory/modulatory way which will dictate the differentiation of naïve T cells into several subsets, e.g., Th1, Th2, Th17, and Treg [96,196]. As mentioned earlier, DCs located at the maternal–fetal interface are faced with two challenges: they must avoid activating T cells specific for inoffensive antigens or risk-inducing autoimmune disease while also being alert to pathogens so that they can prime T cells when needed. This dichotomy is exacerbated during pregnancy since DCs must protect against infection while also being compliant with the novel antigens expressed by the placenta.

When the semi-allogeneic trophoblast cells of the placenta invade through the epithelium and mucosa into the endometrium/decidua, the decidual DCs can process and present fetal-derived trophoblast antigens to the maternal immune system [197]. A way to prevent anti-fetal/placental T cell activation shown in mice is limited migration of DCs to the lymph nodes. The formation of the decidua creates an environment prohibitive of the migration of the tissue-resident DCs out of the decidua and thus promotes immunological tolerance of the fetus [33].

The actions of DCs in pathological pregnancies are predominantly indirect through interactions with cells of the adaptive immune system. For example, DCs have been shown to interact with and cause the proliferation of NKT cells, disrupting the tolerance required during pregnancy and prematurely initiating an inflammatory cascade that leads to labor [198]. In vitro studies have shown that DCs of patients with PE have a more potent ability to induce CD4^+^ T cell differentiation, which could be partially responsible for systemic inflammation in pregnancy complications [199,200].

Since MΦ does not function in isolation, it is possible that their dysregulated activation by other cell types, such as Tregs, could also lead to adverse pregnancy outcomes such as RSA [201].

γ/δ T cells are a subset of T cells involved in inflammatory and immune responses and play a role in linking the innate and adaptive immune systems. γ/δ T cells generally are cytokine-producing cells that create a decidual environment to tolerate the fetus by either secreting IL-10 and TGF-β to inhibit cytotoxic T cells, NK cells, MΦ, DCs, and B cells or by priming Th0 to differentiate into Th cells that suppress effector cells [202,203,204]. In normal pregnancies, they are primarily found in the progesterone receptor-positive/activated form (following trophoblast fetal-antigen recognition) in the peripheral blood of pregnant patients with a bias toward γ1.4/δ1 chain combination [205,206]. Studies have suggested that patients with an increase in the percentage of γ1.4/δ1 cells had significantly decreased NK cell activity, with the above benefits [206]. A few studies found that Vδ2^+^ T cells in patients with PE have increased cytotoxic potential and are less susceptible to apoptosis compared to the same subpopulation from healthy pregnant people [204,207].

## 5. Challenges and Future Perspectives

**Figure 1:**
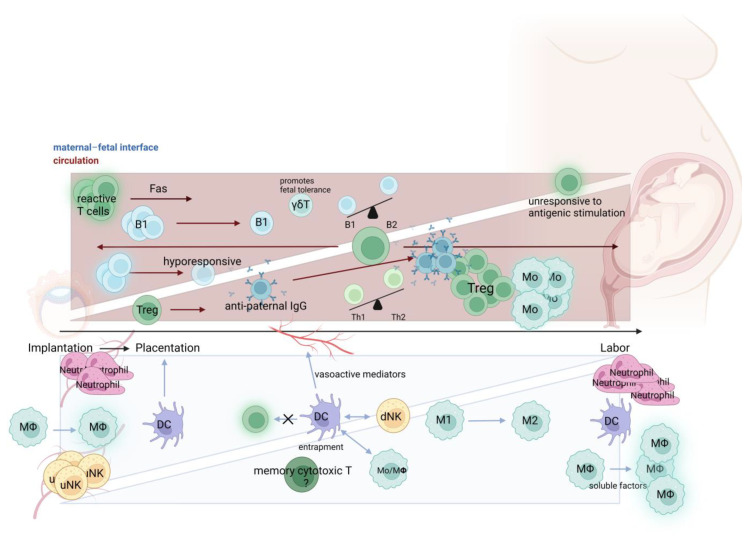
Innate and adaptive immune cells in physiological pregnancy. Schematic representation of immune changes in relation to their localization (i.e., maternal–fetal interface = blue; maternal circulation = red) throughout pregnancy, namely from implantation (far left), placentation to labor (far right). Within the maternal circulation, overall T cells do not significantly change over gestation, but there are changes in T cell subsets; reactive T cells are decreased during pregnancy through FAS-mediated death initiated by trophoblasts, whereas the proportion of regulatory T cells (Treg) is elevated and a Th2 bias is observed. Additionally, there is a highly differentiated CD8^+^ effector memory cell sub-type present at the human decidua with unclear function as denoted by the question mark. B cells are primarily hyporesponsive and present a predominant B2 bias vs. B1 cells. At the maternal–fetal interface, neutrophils and uterine NK cells are highly involved in angiogenesis and vascular remodeling early in pregnancy. Macrophages are activated early in pregnancy, polarize from M1 to M2 mid-gestation, and are again activated in great numbers during labor. DCs interact with macrophages and decidual NK cells; they also inhibit the activation of T cells and are involved early in pregnancy during placentation. The number of neutrophils increase in infiltration of the maternal–fetal interface at the time of labor. Created with BioRender.com (accessed on 16 January 2023).

**Figure 2:**
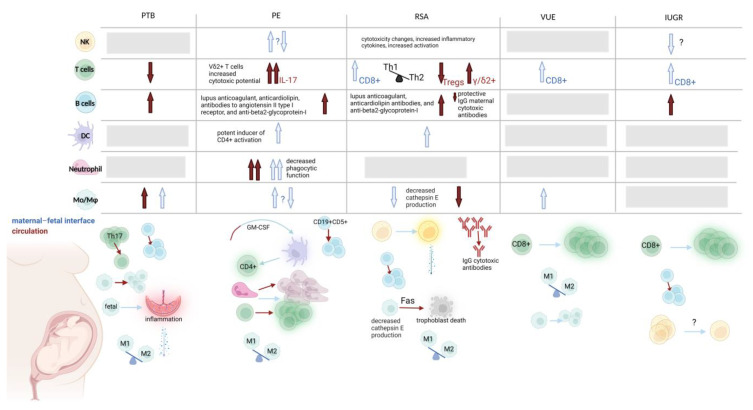
Innate and adaptive immune cells in pathological pregnancy. Changes related to specific cell types in the following pathological pregnancies: preterm birth (PTB), preeclampsia (PE), recurrent spontaneous abortion (RSA), villitis of unknown etiology (VUE), and intra-uterine growth restriction (IUGR). Reported changes in the maternal circulation are presented with red arrows, whereas changes at the maternal–fetal interface are in blue. Grey squares/question marks: unknown/no consensus found in the literature. PTB: Th17 cells are decreased whilst monocytes/macrophages and B cells are increased in the maternal circulation. Fetal macrophages are thought to be involved in placental inflammation. There is a pro-M1 polarization bias at the maternal–fetal interface. PE: DCs activate CD4^+^ T cells and circulating DCs are activated by GM-CSF and infiltrate the maternal–fetal interface. Although neutrophils increase in number, they have decreased phagocytic function in maternal circulation and at the maternal–fetal interface. T cells in general are increased in the maternal circulation and have increased cytotoxic potential. There is also an M1 polarization bias. RSA: NK cells have increased cytotoxicity in the maternal–fetal interface. There are increases in B cells in the maternal circulation and decreased protective IgG maternal cytotoxic antibodies. There is decreased cathepsin E production in macrophages at the decidua which are also involved in Fas-mediated apoptosis of trophoblasts. There is also a skewed M1/M2 macrophage population in the decidua; low circulating levels of monocytes is also indicative of RSA. VUE: CD8^+^ T cells increase in number and in their activation status. Both M1 and M2 macrophages are elevated with a predominance of M1. IUGR: CD8^+^ T cells increase in number and are found to be activated at the maternal–fetal interface. There is conflicting data on decreases in NK cells at the maternal–fetal interface. There is an increase in B cells in the maternal circulation. Created with BioRender.com (accessed on 16 January 2023).

Fully understanding the immune system components involved in pregnancy, is difficult and we attempted to provide a summary of the main roles in physiological (Figure 1) and pathological (Figure 2) pregnancies. Firstly, there are logistical and ethical challenges in obtaining tissues from pregnancies, especially from the first trimester. Secondly, even with animal models of placentation, there are still significant differences compared with human placentation, such as different modes of implantation and limited trophoblast invasion in mouse models [208]. Thus, data obtained from animal models, as always, should be cautiously approached in interpreting physiological or pathological pregnancy. Using explants to organotypically culture cells from a piece or pieces of tissue or organ is one ex vivo accessible method to overcome clinical restrictions of working with the intact complexity of the organ; however, these have limitations as well [209].

More information is also needed on the inflammatory dysregulation at the maternal–fetal interface and in the other compartments, including but not limited to the decidua, amnion, chorion, and overall fetal membranes. For example, Couture et al. have shown evidence of proinflammatory differences in transcriptomes in the placenta, as well as proinflammatory imbalance in fetal membranes, in preterm vs. term pregnancies [210,211]. Localizing inflammatory pathways and understanding the interplay between and the unique contribution of each compartment in physiological or pathological pregnancy is vital in developing efficient therapeutic strategies. Furthermore, as highlighted by this review, there are still conflicting studies on the role of specific immune cell types. To assist in resolving conflicting studies on the role of immune cells, single-cell sequencing studies can provide detailed information regarding the changes in cell types and profile in pathological vs. physiological tissues. This technology provides a novel mean to dissect the cell types and pathways involved in pregnancy, especially the contribution of the immune system [212,213,214]. Recent single-cell level studies have found NK-cell and activated T-cell signatures increased with labor at term, whereas macrophage/monocyte and activated T-cell signature with preterm labor [215]. Even so, much remains to be studied on the single-cell level across gestation in physiological and pathological pregnancies.

More data is needed to answer the following questions: (i) what are the roles of each immune cell population, especially in physiological pregnancy; (ii) what is the contribution to the inflammatory profiles of immune cells at the maternal–fetal interface; (iii) how does the balance or imbalance of immune cell populations correlate with pregnancy complications/outcomes, and health of the neonate; (iv) whether dysregulation of the immune system a contributor or a consequence of adverse pregnancy outcomes, and; (v) what is the interplay of innate and adaptive immune systems throughout pregnancy.

Addressing these key biological questions can also guide the development of novel therapeutics in modulating immune cell subpopulations or targeting secreted mediators. Preclinical studies have successfully rescued mouse fetuses from inflammation-driven fetal death with the adoptive transfer of IL-10-producing B cells or the adoptive transfer of homeostatic macrophages [51,216]. Particulate systems, such as liposomes, polymeric micelles, and polymeric micro- and nanoparticles, efficiently deliver therapeutic agents to MΦ in different physiological portals of entry [217]. The adoptive transfer of syngeneic DCs in murine models has also decreased spontaneous abortions [218]. Other groups have obstructed the maturation of DCs with various chemical, biological, and cellular methods, such as rapamycin, heme oxygenase-1, and mesenchymal stem cells. The addition of these modulators has been found to decrease spontaneous abortion in rodent models [219,220,221]. Further work needs to be carried out on the adoptive transfer of cells and modulating the maturation of immune cells to combat the dysregulation of immune cells in combination with targeting secreted mediators. Interestingly, besides the function of lung maturation of the fetus, maternal corticosteroid administration of betamethasone alters the rate of spontaneous neutrophil apoptosis in early-onset PE [222]. These novel therapeutics and/or different perspectives of existing therapeutics will offer new options for the optimal health of the pregnant individual and fetus. Hormonal control of the maternal immune system also needs to be taken into account, as reviewed here [23,24,25], as some specific cell subtypes can be altered such as antigen presentation by ILCs [223].

The immune system(s) in reproduction cannot be evaluated in isolation, and innate and adaptive immune cell types continue to give each other feedback over time. Although there is not a comprehensive study on interactions between the innate and adaptive immune systems, those interactions are robust and, at this time, can only be studied on a cell (and cell function) basis. Thus, more work must be carried out in order to integrate these two immune systems beyond cell types.

## Data Availability

Not applicable.
